# Parasitic Nematode-Induced CD4^+^Foxp3^+^T Cells Can Ameliorate Allergic Airway Inflammation

**DOI:** 10.1371/journal.pntd.0003410

**Published:** 2014-12-18

**Authors:** Shin Ae Kang, Mi-Kyung Park, Min Kyoung Cho, Sang Kyun Park, Min Seong Jang, Bo-Gie Yang, Myoung Ho Jang, Dong-Hee Kim, Hak Sun Yu

**Affiliations:** 1 Department of Parasitology, School of Medicine, Pusan National University, Yangsan, Republic of Korea; 2 Immunoregulatory therapeutics group in Brain Busan 21 project, Yangsan, Republic of Korea; 3 Academy of Immunology and Microbiology (AIM), Institute for Basic Science (IBS), Pohang, Republic of Korea; 4 Department of Nursing, College of Nursing, Pusan National University, Yangsan, Republic of Korea; Uniformed Services University of the Health Sciences, United States of America

## Abstract

**Background:**

The recruitment of CD4^+^CD25^+^Foxp3^+^T (T_reg_) cells is one of the most important mechanisms by which parasites down-regulate the immune system.

**Methodology/Principal Findings:**

We compared the effects of T_reg_ cells from *Trichinella spiralis*-infected mice and uninfected mice on experimental allergic airway inflammation in order to understand the functions of parasite-induced T_reg_ cells. After four weeks of *T. spiralis* infection, we isolated Foxp3-GFP-expressing cells from transgenic mice using a cell sorter. We injected CD4^+^Foxp3^+^ cells from *T. spiralis*-infected [Inf(+)Foxp3^+^] or uninfected [Inf(-)Foxp3^+^] mice into the tail veins of C57BL/6 mice before the induction of inflammation or during inflammation. Inflammation was induced by ovalbumin (OVA)-alum sensitization and OVA challenge. The concentrations of the Th2-related cytokines IL-4, IL-5, and IL-13 in the bronchial alveolar lavage fluid and the levels of OVA-specific IgE and IgG1 in the serum were lower in mice that received intravenous application of Inf(+)Foxp3^+^ cells [IV(inf):+(+) group] than in control mice. Some features of allergic airway inflammation were ameliorated by the intravenous application of Inf(-)Foxp3^+^ cells [IV(inf):+(-) group], but the effects were less distinct than those observed in the IV(inf):+(+) group. We found that Inf(+)Foxp3^+^ cells migrated to inflammation sites in the lung and expressed higher levels of T_reg_-cell homing receptors (CCR5 and CCR9) and activation markers (Klrg1, Capg, GARP, Gzmb, OX40) than did Inf(-)Foxp3^+^ cells.

**Conclusion/Significance:**

*T. spiralis* infection promotes the proliferation and functional activation of T_reg_ cells. Parasite-induced T_reg_ cells migrate to the inflammation site and suppress immune responses more effectively than non-parasite-induced T_reg_ cells. The adoptive transfer of Inf(+)Foxp3^+^ cells is an effective method for the treatment and prevention of allergic airway diseases in mice and is a promising therapeutic approach for the treatment of allergic airway diseases.

## Author Summary

Many studies have investigated the down-regulation of the immune system by parasite infection. CD4^+^CD25^+^Foxp3^+^T (T_reg_) cells are key players in parasite-mediated immune downregulation. Our previous study suggested that T_reg_ cells recruited by *Trichinella spiralis* infection were the key cells mediating the amelioration of allergic airway inflammation in mice. In the present study, we investigated the functions of parasite-induced T_reg_ cells using mice expressing GFP-tagged Foxp3. *T. spiralis* infection increased the number of T_reg_ cells. Adoptive transfer of the parasite-induced T_reg_ cells to mice with allergic airway inflammation ameliorated allergic airway inflammation. The transferred cells were recruited to inflammation sites in the lung. Cells from parasite-infected mice expressed higher levels of T_reg_-cell homing receptors and activation markers than did cells from uninfected mice. This study might help explain why immune disorders (often of unknown cause) are more prevalent among people in developed countries (areas with low parasite infection) than among those in developing countries (areas with parasite epidemics). Our finding might improve current cell therapy techniques and facilitate the development of new techniques that use parasites or parasite-borne materials to treat diverse immune disorders.

## Introduction

In humans, trichinellosis, caused by oral infection with *Trichinella* sp., is typified by an intestinal phase and a muscular phase, corresponding to two distinct periods in the parasite's life cycle in the host [1], [2]. The physiopathological symptoms include heavy muscle aches, fever, and eosinophilia [3]. During each of the two phases, the host immune system activates different responses to the infection. Th2-related cytokine levels increase immediately after *T. spiralis* larvae invade the intestine [4], and the levels of IL-4 and IL-13 peak before the initiation of nurse cell formation [4], [5]. Additionally, the levels of most Th17-related cytokines increase until the muscle phase begins. Th2- and Th17-related cytokine levels decrease after the recruitment of CD4^+^CD25^+^ Forkhead box P3 (Foxp3)^+^T (T_reg_) cells to the spleen and lymph nodes [4]. T_reg_ cells appear to play a role in the maintenance of chronic infections or in the suppression of the parasite targeting immune response [4], [6].

T_reg_ cells contribute to the maintenance of host immune homeostasis by actively suppressing various pathological and physiological immune responses [7]. To reduce the infectious burden, parasites can influence natural T_reg_ cells by modifying the T-cell immune response at the infection site, thus allowing the parasite to survive in the host for longer periods [8]. Although some controversy remains, two different mechanisms are thought to underlie the suppression of T_reg_ cells during parasite infection. In the first, the interaction of the T effector ligands CD80 and CD86 with cytotoxic-T-lymphocyte-associated protein (CTLA-4) activates the transmission of immunosuppressive signals on T effector cells, thereby reducing the function of effector T-cells. In the second, cytokines such as IL-10 and transforming growth factor (TGF-β) mediate suppression [8], [9]. After some parasite infections, T_reg_ cells activate specific genes, such as those encoding CD103, Foxp3, glucocorticoid-induced TNFR family related gene (GITR), OX40 (CD134), CTLA-4, secretory leukocyte peptidase inhibitor (Slpi), granzyme B (Gzmb), fatty acid-binding protein 5 (Fabp5), nuclear factor, interleukin 3 regulated (Nfil3), suppressor of cytokine signaling 2 (Socs2), G protein-coupled receptor 177 (Gpr177), and killer cell lectin-like receptor subfamily G, member 1 (Klrg1) [10]–[14]. However, the roles and mechanisms of T_reg_ cell-mediated suppression remain controversial and require further investigation [15]. Although many studies have demonstrated that parasites can activate and induce the T_reg_-cell population, few studies have investigated the immune regulatory mechanisms of parasite-induced T_reg_ cells after their direct transfer into animals with immune disorders. The OVA-alum allergic airway inflammation model has been widely used as an animal model of immune disorders because it enables the study of Th2-mediated allergic responses [16]–[19].

In a previous study, we observed that *T. spiralis* infection induced the T_reg_-cell population and increased IL-10 and TGF-β cytokine levels, and infection may also reduce artificially induced allergic airway inflammation [20]. In this study, to examine the functional roles of parasite-induced T_reg_ cells, we evaluated the expression of T_reg_-cell surface markers (related to homing, suppression ability, and responses to inflammatory cytokines) and the functional effects of induction with *T. spiralis*. In addition, we intravenously injected parasite-induced CD4^+^Foxp3^+^T cells and natural CD4^+^Foxp3^+^T cells into normal mice before and during airway inflammation.

## Methods

### Parasites

The *T. spiralis* strain (isolate code ISS623) was maintained in our laboratory by serial passage in rats. Carcasses of infected mice were eviscerated and cut into pieces. The parasite-infected muscles were digested in 1% pepsin-hydrochloride solution with constant stirring for 1 h at 37°C. The muscle-stage larvae were collected under a microscope after removal of the pepsin-hydrochloride solution. The larvae were rinsed more than 10 times in sterile PBS.

### Preparation of GFP-expressing CD4^+^Foxp3^+^ T cells

During the experimental period, Foxp3-eGFP mice (expressing GFP-tagged Foxp3) purchased from Jackson Laboratory were maintained in a specific pathogen-free facility at the Institute for Laboratory Animals of Pusan National University. Foxp3^+^ cells were isolated from the spleen of *T. spiralis*-infected [Inf(+)Foxp3^+^] and uninfected [Inf(-)Foxp3^+^] Foxp3-eGFP mice. The spleens were minced into small pieces, which were placed into ACK hypotonic lysis solution (Sigma, USA) at room temperature for 2 min to lyse erythrocytes (red blood cells, RBCs). Following lysis, the remaining cells were filtered through 100-µm meshes (Small Parts, Inc., USA) and washed three times. CD4^+^ T cells were isolated using a CD4^+^ T cell isolation kit (Miltenyi Biotech, USA) in accordance with the manufacturer's protocol. Foxp3^+^ (GFP^+^) cells were obtained using a FACS cell sorter.

### Cell transfer to allergic airway inflammation-induced mice

**Figure 1:**
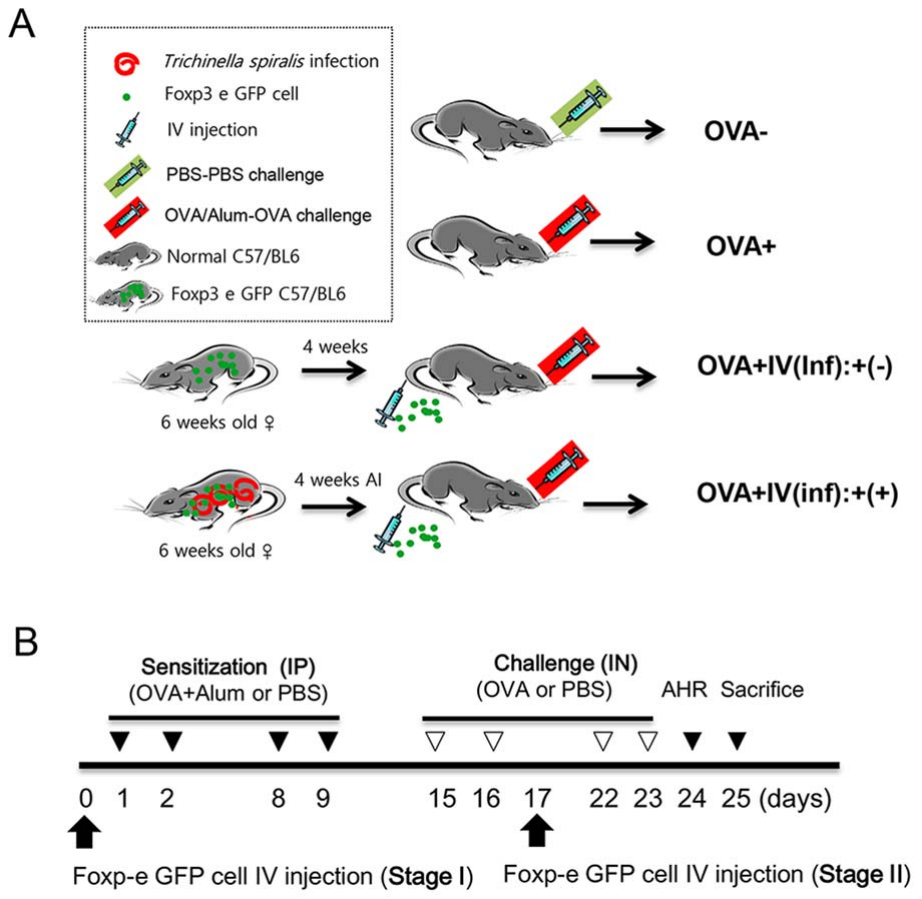
Experimental protocol of T_reg_ cell adoptive transfer and the airway inflammation induction model. The mice were divided into four experimental groups based on allergy induction and the adoptive transfer of T_reg_ cells isolated from *T. spiralis-infected* mice or uninfected mice. After four weeks of *T. spiralis* infection, T_reg_ (CD4^+^Foxp3^+^T) cells were isolated from spleen using a FACS sorter (A). Airway inflammation was induced via OVA-Alum or PBS sensitization and OVA or PBS challenge according to the experimental protocols given in the Methods (B). (OVA-; PBS treated group, OVA+; allergic airway inflammation-induced group, OVA+IV(Inf)+(-); allergic airway inflammation-induced and CD4^+^Foxp3^+^T cell of normal mice adoptive transferred group, OVA+IV(Inf)+(+); allergic airway inflammation-induced and CD4^+^Foxp3^+^T cell of *T. spiralis-infected* mice adoptive transferred group. AI; after infection, IP; intra peritoneal, IN, intra nasal, IV; intra venous).

Five-week-old female C57BL/6 mice were purchased from Samtako Co. (Korea). Four groups of mice were used. In the first group of mice, allergic airway inflammation was induced via intraperitoneal (IP) injection of ovalbumin (OVA)-alum for sensitization followed by intranasal (IN) challenge with OVA four times, without adoptive cell transfer [OVA+]. The second group of mice was injected (IP) and challenged (IN) with PBS, without adoptive cell transfer [OVA-]. The third group of mice was administered Inf(+)Foxp3^+^ cells (5 × 10^5^) intravenously, and allergic airway inflammation was induced with OVA/alum injection (IP) and OVA challenges (IN) [OVA+IV(inf):+(+) group]. The fourth group of mice was administered Inf(-)Foxp3^+^ cells, following the same protocol used for the third group of mice [OVA+IV(inf):+(-) group] (Fig. 1A). To evaluate the efficiency of the adoptive transfer according to the injection time, we transferred cells before the allergic airway inflammation induction period (Stage I, preventive effect) or after the first allergen challenge (Stage II, therapeutic effect). Allergic airway inflammation was induced as previously reported with some modifications [20]. Briefly, mice were sensitized via IP injection of 75 µg OVA (Sigma-Aldrich, USA) and 2 mg of aluminum hydroxide (alum; Sigma-Aldrich) in 200 µL of 0.9% sterile saline on days 1, 2, 8, and 9. The mice were then challenged with IN administration of 50 µg of OVA on days 15, 16, 22, and 23. Airway hyper-responsiveness (AHR) was measured on day 24, and the mice were sacrificed on day 25 (Fig. 1B).

### Lymphocyte preparation

Lymphocytes were isolated from the spleen and lung-draining lymph nodes (LLN) of mice to determine the levels of allergen (OVA)-specific, cytokine-secreting lymphocytes. The methods were identical to those described above for the preparation of CD4^+^Foxp3^+^ T cells. The isolated cells were plated onto OVA-coated wells and non-coated wells at 5×10^6^ cells/mL in RPMI 1640 with 10% fetal bovine serum and penicillin/streptomycin. After incubation for 72 h at 37°C in 5% CO_2,_ culture supernatants was harvested and stored at −20°C for ELISA.

### Analysis of bronchoalveolar lavage fluid (BALF)

After mice were anesthetized, the tracheas were exposed and cut just below the larynx. A flexible polyurethane tube attached to a blunt 24-gauge needle, with a 0.4-mm outer diameter and length of 4 cm (Boin Medical Co., Korea), 800 µL of cold PBS was inserted into the trachea. The BALF samples were collected and centrifuged for 5 min at 1500 rpm at 4°C. After centrifugation, the supernatants were collected and quickly frozen at −70°C. The cell pellets were resuspended in 100 µL of ACK hypotonic lysis solution (Sigma) and incubated for 2 min to lyse the RBCs. Next, 900 µL of PBS was added, and the cell suspension was centrifuged for 5 min at 3000 rpm at 4°C. The supernatants were then decanted, and the cell pellets were resuspended in 100 µL of PBS. After each procedure, the cells were centrifuged onto microscope slides for 5 min at 500 rpm using a Cytospin apparatus (Micro-12TM; Hanil Co., Korea). The microscope slides were air-dried and stained with Diff-Quik (Sysmex Co., Japan). Cells on the stained slides were counted in a blinded manner under a light microscope. At least 500 cells were counted per slide.

### AHR measurements

At 24 h after the last allergen challenge, airway responsiveness was evaluated by measuring the change in lung resistance in response to aerosolized methacholine (Sigma) [21]. To measure bronchoconstriction, the enhanced pause (PenH) was measured at baseline (PBS aerosol) and after exposure to increasing doses of aerosolized methacholine (0–50 mg/mL) using whole-body plethysmography (Allmedicus, Korea). In the plethysmography procedure, the mice were acclimated for 3 min, exposed to nebulized saline for 10 min, and treated with increasing concentrations (0, 12.5, 25, and 50 mg/mL) of methacholine using an ultrasonic nebulizer (Omron, Japan). After each nebulization, the PenH values measured every three minutes during the experimental period were averaged. Graphs were generated showing the PenH values in response to increasing methacholine concentrations for each dose-matched group of mice.

### Lung histopathology studies

Histopathological analyses were performed as described previously [22]. In brief, lung tissues were fixed in a 10% formaldehyde solution and embedded in paraffin. The tissue was then cut into sections, and the sections were stained with hematoxylin-eosin (H&E) and periodic acid-Schiff (PAS) stains. The stained sections were evaluated under a microscope [23].

### Total RNA extraction and real-time PCR

Total RNA was extracted from the lung using 1 mL of BIOZOL (LPS Solution, Korea), and cDNA was synthesized using MMLV reverse transcriptase (Promega, USA) according to the protocols provided by the manufacturer. MUC2, MUC5, and eotaxin RNA levels were quantified using a real-time PCR machine (Bio-Rad Laboratories, Inc., USA) according to the manufacturer's instructions. Total RNA was extracted from sorted Foxp3+ cells. The transcript levels of chemokine (C-X-C motif) receptor 3 (CXCR3), chemokine (C-C motif) receptor (CCR) 4, CCR5, CCR9, CCR10, Klrg1, capping protein gelsolin-like (Capg), Gzmb, glycoprotein A repetitions predominant (GARP), CTLA-4, CD62L, and OX40 in the T_reg_ cells were analyzed using real-time PCR. The primer sequences are listed in S1 Table. The relative expression of each gene was calculated as the ratio of target gene expression to housekeeping gene (*GAPDH*) expression using the Gene-x program (Bio-Rad laboratories, Inc.).

### ELISA of immunoglobulin (Ig) and cytokine levels

After mice were sacrificed, serum was collected via cardiac puncture. The serum was diluted 1∶40 (for IgG1 and IgG2a) in blocking buffer. OVA-specific IgG1, IgG2a, and IgE levels in the serum and cytokine [IL-4, IL-5, IL-10, IL-13, interferon (IFN)-γ, TGF-β] levels in the BALF and culture supernatants of LLN were measured using ELISA in accordance with the manufacturer's instructions (eBioscience, USA). The absorbance of the final reactant was measured at a wavelength of 450 nm with an ELISA plate reader.

### Flow cytometry

To evaluate the recruitment of T_reg_ cells after the adoptive transfer of Foxp3^+^ cells, live cells were isolated from the spleen and LLN of OVA-induced allergic airway inflammation mice that had been infected or not infected with *T. spiralis*. The cell preparation method was identical to those of section “lymphocyte preparation”. The samples were acquired using a FACS maschine. The following mAbs were used: anti-CD4-PE, anti-CD25-APC, anti-CD39-efluor 660, and anti-CTLA-4-PE.

### Immunohistochemistry and confocal microscopy

Paraffin sections of lung tissue were deparaffinized and then treated with an antigen retrieval solution for 20 min (0.1 M citric acid, 0.1 M sodium citrate, pH 6.0). The slides were rinsed with PBS and immersed in methanol (0.3% H_2_O_2_) for 15 min to inhibit endogenous peroxidase activity. After pre-incubation with 1% BSA for 1 h at room temperature, the sections were incubated with hamster anti-mouse CTLA-4 (1∶500; Santa Cruz Biotechnology, USA) for 1 h at 4°C. After several washes in PBS, the Alexa Fluor 594 goat anti-hamster IgG secondary antibody (1∶500; Jackson ImmunoResearch Laboratories, USA) was applied for 1 h at room temperature. The slides were washed in PBS and incubated with DAPI for 2 min. Confocal images of stained lung tissue or stained specific T_reg_ cells were examined under an inverted fluorescence microscope.

### T-cell proliferation inhibition assay

In 96-well round-bottomed plates, purified splenocytes were cultured in the presence of 1 µg/mL anti-CD3. The number of responder splenocytes per well was kept constant at 3×10^4^ cells, while the number of suppressor cells was varied. Normal splenocytes were mixed at several different ratios with CD4^+^Foxp3^+^ cells isolated from parasite-infected mice or uninfected mice in 200 µL of complete medium. Cell viability analysis using the trypan blue dye exclusion assay was performed three days later. After trypsinization, the number of viable cells in triplicate wells at each concentration was estimated using a hemocytometer. The cells in each well were counted three times, and the experiment was repeated three times [24].

### Statistical analysis

Data were analyzed using SPSS for Windows, version 14 (SPSS, USA). Student's *t*-test or ANOVA was used to compare the group means.

### Ethics statement

The study was performed with approval from the Pusan National University Animal Care and Use Committee (Approval No. PNU-2013-0293), in compliance with “The Act for the Care and Use of Laboratory Animals” of the Ministry of Food and Drug Safety, Korea. All animal procedures were conducted in a specific pathogen-free facility at the Institute for Laboratory Animals of Pusan National University.

## Results

### Adoptive transfer of parasite-induced CD4^+^Foxp3^+^ cells inhibits OVA-induced airway inflammation

**Figure 2:**
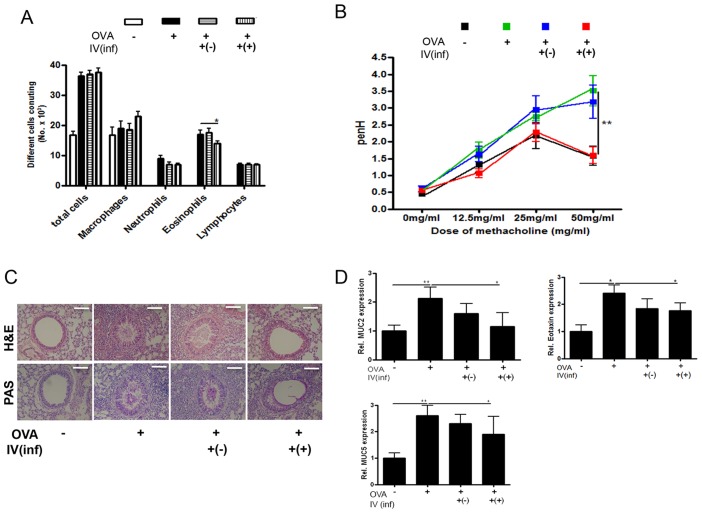
Amelioration of airway inflammation by CD4^+^Foxp3^+^T cell adoptive transfer before asthma induction (Stage I). The number of inflammatory cells in the BALF samples was counted after Diff-Quik staining (A). The enhanced pause (PenH) was evaluated at baseline and after treatment with increasing doses of aerosolized methacholine (0–50 mg/mL). square; control mice, triangle; Ova-Alum treated mice, inverted triangle; OVA-Alum treated mice that receive T_reg_ cells from uninfected mice, diamond; OVA-Alum treated mice that receive T_reg_ cells from *T.spiralis* infected mice (B). The histological appearance of lungs after challenge with OVA and cell transfer (bar = 100 µm). The thin sections of lung were then stained with hematoxylin-eosin (H&E) and PAS stains (C). Relative quantification of *MUC2 MUC5* and *eotaxin* gene expression in lung after the induction of airway inflammation (D). Total RNA was extracted from lung tissue and cDNA was synthesized. The gene expression levels of MUC2, MUC5 and *eotaxin* in the lungs of each group were analyzed using real-time PCR. The GAPDH gene was used as a control. Data are representative of three independent experiments [OVA-; PBS treated mice, OVA+; allergic airway inflammation-induced mice, IV(inf)+(-); CD4^+^Foxp3^+^T cell of normal mice adoptive transferred mice, IV(inf)+(+); CD4^+^Foxp3^+^T cell of *T. spiralis-infected* mice adoptive transferred mice, *p<0.05, **p<0.01, *n* = 6 mice/group].

To determine whether *T. spiralis*-induced T_reg_ cells can prevent allergic airway inflammation in an OVA-alum asthma mouse model, we injected mice with CD4^+^Foxp3^+^ T cells isolated from *T. spiralis*-infected [Inf(+)Foxp3^+^] or uninfected [Inf(-)Foxp3^+^] mice and evaluated allergic inflammation responses after OVA sensitization and challenge (Fig. 1B, Stage I). Fig. 2A shows the changes in the immune cell populations in the BALF of asthma-induced mice after the adoptive transfer of Inf(+)Foxp3^+^ cells. The adoptive transfer of CD4^+^Foxp3^+^ cells from *T. spiralis*-infected mice, but not of those from uninfected mice, reduced the number of eosinophils in the BALF. To evaluate airway function, airway responsiveness was determined after treating the mice with increasing doses of methacholine. Methacholine increased the PenH value in the OVA-induced allergic airway inflammation group in a dose-dependent manner. The introduction of Inf(+)Foxp3^+^ cells decreased the PenH value in OVA-induced mice, whereas Inf(-)Foxp3^+^ cell treatment did not (Fig. 2B).

Following the induction of airway inflammation, an influx of inflammatory cells into the peribronchial spaces was observed. The influx of inflammatory cells led to the destruction of the alveolar wall and generated severe hemorrhage. We observed hypertrophy of goblet cells in the peribronchial epithelium and high amounts of mucus production in the OVA-induced allergic airway inflammation group via PAS staining. In the Inf(+)Foxp3^+^ cell transfer group, inflammatory cell infiltration in the peribronchial areas decreased somewhat, and a small amount of mucus production and decreased goblet cell hyperplasia were observed in the peribronchial epithelia, with little hypertrophy of goblet cells in the tracheal and bronchial epithelia (Fig. 2C). Gene expression related to mucus production (*MUC2* and *MUC5*) and eosinophil chemoattractant (*eotaxin*) in the lung was lower in the Inf(+)Foxp3^+^ cell transfer group than in the OVA-challenged group (Fig. 2D).

### Adoptive transfer of parasite-induced CD4^+^Foxp3^+^ T cells inhibits Th2 cytokine production

**Figure 3:**
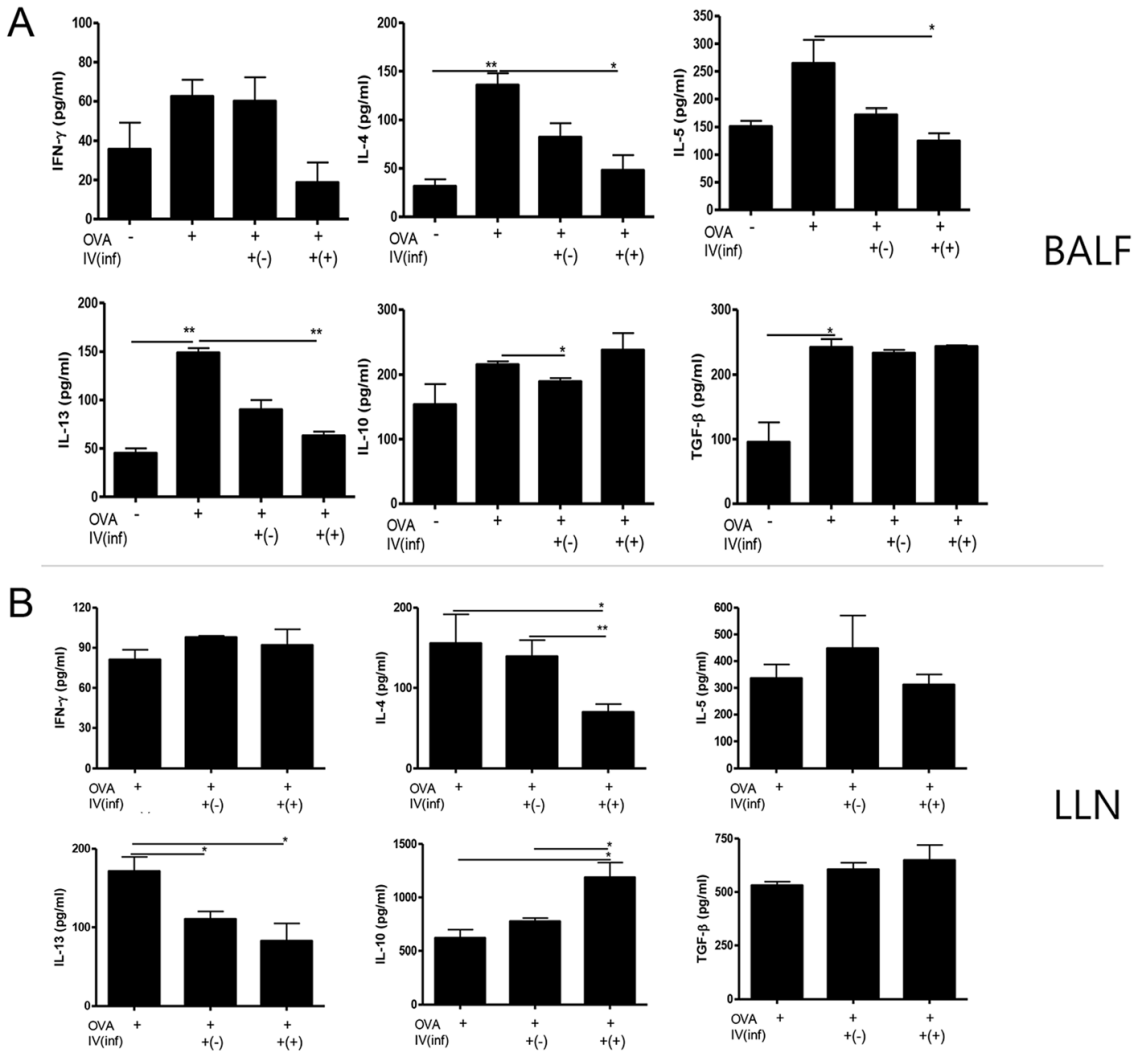
Cytokine concentrations in BALF and OVA specific lymphocytes isolated from lung draining lymph node. Cytokine concentrations were measured in the BALF samples (A). The lymphocytes were activated by OVA. The wells was incubated with 1 µg/mL of OVA for 16 h at 4°C, and then the lymphocytes isolated from lung draining lymph node (LLN) were added to the well and incubated for three days. After activation, cytokine concentrations in the supernatant were measured using ELISA kits, in accordance with the manufacturer's instructions (B). [OVA-; PBS treated mice, OVA+; allergic airway inflammation-induced mice, IV(inf)+(-); CD4^+^Foxp3^+^T cell of normal mice adoptive transferred mice, IV(inf)+(+); CD4^+^Foxp3^+^T cell of *T. spiralis-infected* mice adoptive transferred mice, *p<0.05, **p<0.01, *n* = 6 mice/group, 3 independent experiments].

To characterize the effects of CD4^+^Foxp3^+^ T-cell transfer on cytokine secretion in the BALF, an ELISA was performed to monitor the expression of Th1 (IFN-γ), Th2 (IL-4, IL-5, and IL- 13), and regulatory (IL-10 and TGF-β) cytokines. The concentrations of IL-4, IL-5, and IL-13 in the BALF of the Inf(+)Foxp3^+^ cell transfer group were lower than those in the other OVA-challenged groups (*p*<0.05; Fig. 3A). Inf(+)Foxp3^+^ cell transfer did not affect the production of IL-10 and TGF-β (Fig. 3A). In the LLN, IL-4 and IL-13 cytokine production by lymphocytes decreased in the Inf(+)Foxp3^+^ cell transfer group, but IL-5 production was not affected by the cell transfer. The cytokine profiles of the Inf(-)Foxp3^+^ cell transfer group were mostly similar to those of the Inf(+)Foxp3^+^ cell transfer group. Th2 cytokine production was inhibited by Inf(-)Foxp3^+^ cell transfer, but the effect was less than that seen in the Inf(+)Foxp3^+^ cell transfer group (Fig. 3B).

**Figure 4:**
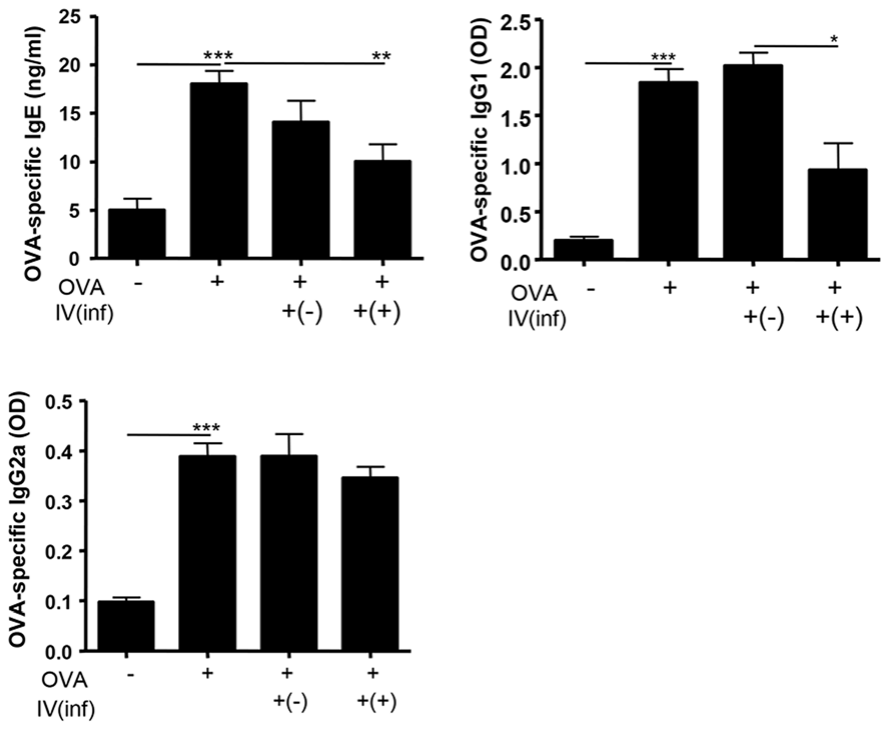
OVA-specific serum immunoglobulin levels. OVA-specific IgE, IgG1, and IgG2a levels in serum were measured. Ninety six well plates were incubated with OVA (final concentration 1 µg/mL) for 16 h at 4°C, and the immunoglobulins levels were measured (the serums were diluted with PBS to 1∶40 for the IgG1 and IgG2a measurements) after a triple wash with PBS. [OVA-; PBS treated mice, OVA+; allergic airway inflammation-induced mice, IV(inf)+(-); CD4^+^Foxp3^+^T cell of normal mice adoptive transferred mice, IV(inf)+(+); CD4^+^Foxp3^+^T cell of *T. spiralis-infected* mice adoptive transferred mice, *p<0.05, **p<0.01, *n* = 6 mice/group, 3 independent experiments].

OVA challenge increased the levels of OVA-specific IgE and IgG1. The increase was attenuated by Inf(+)Foxp3^+^ cell transfer, but not by Inf(-)Foxp3^+^ cell transfer. The level of OVA-specific IgG2a was similar in each group (Fig. 4).

### Transferred CD4^+^Foxp3^+^ cells infiltrate the airway around sites of inflammation

**Figure 5:**
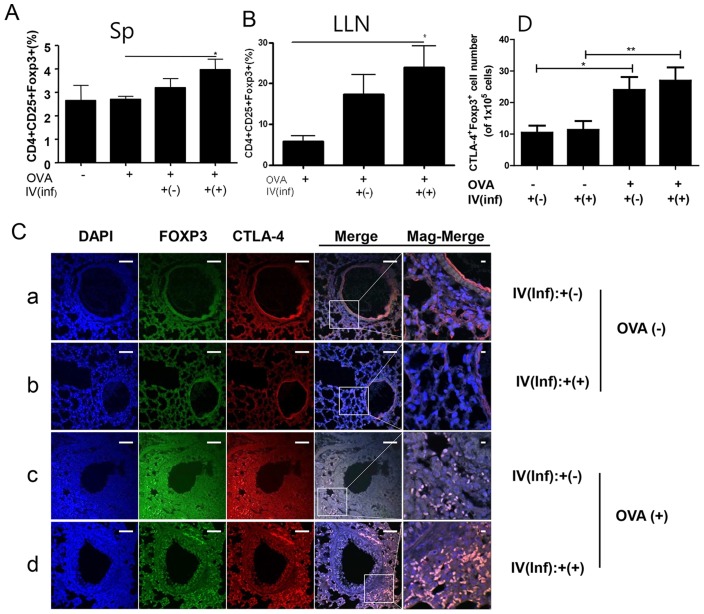
Recruitment of T_reg_ cells into spleen, lung draining lymph node, and the lung of the CD4^+^Foxp3^+^T cell adoptive transferred mice before asthma induction. The lymphocytes were isolated from spleen (A) and lung draining lymph node (B). Paraffin sections of lungs from mice receiving CD4^+^Foxp3^+^T cells (green) were immunofluorescently stained for CTLA-4 (red), and nuclei (DAPI, blue) representative pictures are shown, (white bar = 100 µm) (C). [a; OVA-IV(inf):+(-) group, b; OVA-IV(inf):+(+) group, c; OVA+IV(inf):+(-) group, d; OVA+IV(inf):+(+) group.] Population of CTLA4^+^Foxp3^+^ cells of each group were analyzed in the total lung cells based on analysis the S1B Fig. using Image J program (The value in grape represented the number of the CTLA4^+^ Foxp3^+^ cells per 1×10^5^ DAPI positive cells) (D). [Sp; spleen, LLN; lung draining lymph node, Merge; merge of CTLA-4, GFP, and DAPI filed screens, Mag-Merge; magnification of Merge, OVA-; PBS treated mice, OVA+; allergic airway inflammation-induced mice, IV(inf)+(-); CD4^+^Foxp3^+^T cell of normal mice adoptive transferred mice, IV(inf)+(+); CD4^+^Foxp3^+^T cell of *T. spiralis-infected* mice adoptive transferred mice, *p<0.05, **p<0.01, *n* = 6 mice/group, 3 independent experiments].

To understand the mechanism of reduced airway inflammation in CD4^+^Foxp3^+^T cell-transferred mice, we examined T_reg_ cells in the spleen and LLN. The T_reg_-cell subset increased in the spleen and LLN after Inf(+)Foxp3^+^ cell transfer (Fig. 5A and B). In the LLN, the T_reg_-cell population also increased after Inf(-)Foxp3^+^ cell transfer (Fig. 5B). In addition, *Foxp3* gene expression in the lung increased after OVA challenge. Inf(+)Foxp3^+^ cell transfer further increased *Foxp3* gene expression than only OVA challenge group, whereas Inf(-)Foxp3^+^T cell transfer did not (S1A Fig.).

To determine the origin of the T_reg_ cells in the lung, we examined transferred Foxp-eGFP cells in the lung using confocal microscopy. In addition, we evaluated the activation of T_reg_ cells by examining CTLA-4 expression on Foxp-eGFP cells in lung tissue. Interestingly, we detected Foxp-eGFP cells in the lungs of Inf(+)Foxp3^+^ and Inf(-)Foxp3^+^ cell-transferred mice. In mice without OVA-induced inflammation, a few Foxp-eGFP cells were found in the lung matrix, but not around the airways (Fig. 5C-a & -b and S1B Fig.). However, numerous Foxp-eGFP cells were detected in the allergic airway of inflammation-induced mice, particularly in Inf(+)Foxp3^+^ cell-transferred mice (Fig. 5D-c and 5D-d). Almost all of the Foxp-eGFP cells in the lung strongly expressed CTLA-4, a surface marker for T_reg_-cell activation, and these cells were detected around the sites of airway inflammation (Fig. 5D-c, 5D-d, S1A and B Fig.).

### Adoptive transfer of CD4^+^Foxp3^+^ T cells has a therapeutic effect on airway inflammation

**Figure 6:**
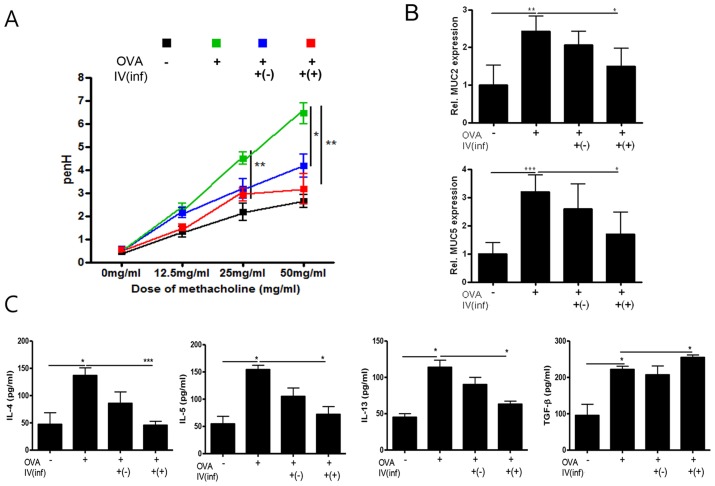
Amelioration of airway inflammation with CD4^+^Foxp3^+^T cell adoptive transfer during asthma induction (Stage II). The PenH values were determined at baseline and after treatment with increasing doses of aerosolized methacholine (0–50 mg/mL) (A). Relative quantification of MUC2 and MUC5 gene expression in lung after induction of airway inflammation (B). Cytokine concentrations were measured in the BALF samples (C).

**Figure 7:**
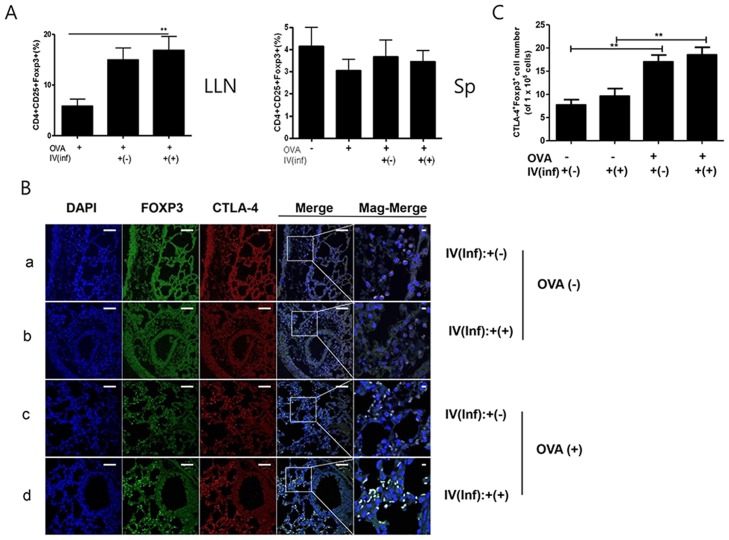
Recruitment of T_reg_ cells into spleen, lung draining lymph node, and lung with CD4^+^Foxp3^+^T cell adoptive transfer during asthma induction in mice. The lymphocytes were isolated from spleen and lung draining lymph node (A). After staining, the lymphocytes were firstly gated with CD4 and the percentage of CD25+Foxp3+ cells calculated using FACS analysis. Paraffin sections of the lung from all of the mice in each group receiving CD4^+^Foxp3^+^T cells (green) were immunofluorescently stained for CTLA-4 (red) and nuclei (DAPI, blue), representative pictures are shown, (white bar = 100 µm) (B). Population of CTLA4^+^Foxp3^+^ cells of each group were analyzed in the total lung cells based on analysis the S4 Fig. using Image J program(C). (The value in grape represented the number of the CTLA4^+^ Foxp3^+^ cells per 1×10^5^ DAPI positive cells) [a; OVA-IV(inf):+(-) group, b; OVA-IV(inf):+(+) group, c; OVA+IV(inf):+(-) group, d; OVA+IV(inf):+(+) group. Merge; merge of CTLA-4, GFP, and DAPI filed screens, Mag-Merge; magnification of Merge, Sp; spleen, LLN, lung draining lymph node, OVA-; PBS treated mice, OVA+; allergic airway inflammation-induced mice, IV(inf)+(-); CD4^+^Foxp3^+^T cell of normal mice adoptive transferred mice, IV(inf)+(+); CD4^+^Foxp3^+^T cell of *T. spiralis-infected* mice adoptive transferred mice, *p<0.05, **p<0.01, *n* = 6 mice/group, 3 independent experiments].

To determine the therapeutic effects of CD4^+^Foxp3^+^ T cells on allergic airway inflammation, we introduced two types of CD4^+^Foxp3^+^ T cells at the initiation of inflammation and investigated the disease index of allergic airway inflammation, as in the Stage I experiment (Fig. 1B, Stage II). The introduction of Inf(+)Foxp3^+^ cells ameliorated airway inflammation: airway responsiveness values, mucin secretion in the airway, and *MUC2*, *MUC5*, and *eotaxin* gene expression in the lung were reduced (Fig. 6A and 6B and S2A & S2B Fig.). However, although the number of eosinophils was reduced in the BALF, the change was not significant (S2C Fig.). The Th2 cytokine concentration in the BALF of Inf(+)Foxp3^+^ cell-transferred mice decreased, but the concentration of TGF-β increased (Fig. 6C). IFN-γ and IL-10 concentrations were not affected by Inf(+)Foxp3^+^ cell transfer (S3A Fig.). Except for IL-4, Th2 and regulatory cytokine production in lymphocytes isolated from the LLN after CD4^+^Foxp3^+^ T-cell transfer did not change (S3B Fig.). The T_reg_-cell population increased in the LLN, but not the spleen, of Inf(+)Foxp3^+^ cell-transferred mice (Fig. 7A). As with the Stage I experiment, we detected a few Foxp-eGFP cells in the lungs of mice in which inflammation was not induced (Fig. 7B-a and 7B-b). In the Stage II experiment, many Foxp-eGFP cells were detected in mice with allergic airway inflammation, but in the microscopy fields examined, there appeared to be fewer CTLA-4-expressing cells (Ave. 18.45 cells/10^5^ DAPI^+^ cell) than those (Ave. 28.95 cells/10^5^ DAPI^+^ cell) in the Stage I experiment (Fig. 5D & 7C).

### *T. spiralis* infection enhances the immune regulatory abilities of T_reg_ cells

**Figure 8:**
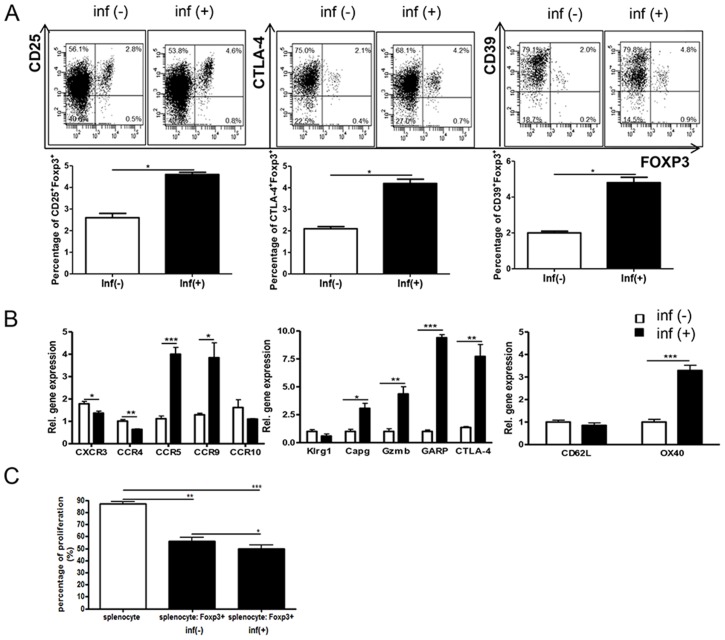
Characterization of parasite-induced CD4^+^Foxp3^+^T cells. Splenocytes from *T. spiralis-infected* Foxp3 e GFP mice and normal Foxp3 e GFP mice. The GFP^+^ population was sorted using a FACSAria. After staining, the plots indicate the expression levels of several surface markers (related to T_reg_ function and activation) in gated CD4+ cell. The graphs showed mean value of the experimental group (A). Total RNA was extracted from Foxp3^+^ cells and cDNA was synthesized. The various gene expression levels in the lungs of each group were analyzed using real-time PCR. The GAPDH gene was used as a control. The expression of the important homing receptors, which are activation markers of T_reg_ cells, were analyzed (B). Purified splenocytes were cultured in 96-well round-bottomed plates in the presence of 1 µg/mL anti-CD3. Normal splenocytes were mixed in a 8∶1 ratio with CD4^+^Foxp3^+^T cells of parasite-infected and normal mice, in 200 µL of complete medium. After three days, cell viability was determined by trypan blue dye exclusion assay (C). (*p<0.05, **p<0.01, *** *p*<0.001, *n* = 5 mice/group, three independent experiments).

To evaluate changes in the molecular characteristics of T_reg_ cells following *T. spiralis* infection, we analyzed surface markers on T_reg_ cells isolated from the spleen. The number of CD4^+^CD25^+^Foxp3^+^T cells and CD4^+^CD25^-^Foxp3^+^T cells increased following *T. spiralis* infection. In addition, the T_reg_-cell activation markers CTLA-4 and CD39 significantly increased after parasitic infection (Fig. 8A). In addition, the expression of *CCR5* and *CCR9*, which encode T_reg_-cell homing receptors, was higher than in Inf(-)Foxp3+ cells, whereas the expression of *CXCR3* and *CCR4* in Inf(+)Foxp3^+^ cells was lower (Fig. 8B). We analyzed the expression of genes related to the function and activation of T_reg_ cells, such as *Klrg1, Capg, GARP, Gzmb*, and *OX40*. Except for *Klrg1*, the expression of these genes in Inf(+)Foxp3^+^ cells was 3- to 10-fold higher than in Inf(-)Foxp3^+^ cells (Fig. 8B). We compared the functional properties of Inf(+)Foxp3^+^ cells and Inf(-)Foxp3^+^ cells using naive T-cell co-culture. Both types of T_reg_ cells efficiently inhibited T-cell proliferation. However, Inf(+)Foxp3^+^ cells inhibited T-cell proliferation more effectively than did Inf(-)Foxp3^+^ cells (50.5% vs. 56.1%, respectively) (Fig. 8C).

## Discussion

To maintain their long-term survival in a host organism, helminthic parasites have immune suppressive abilities that can modulate the host immune response [7]. The immune-modulating functions of helminthic parasites are used in the treatment of several immunological diseases, including inflammatory bowel disease, autoimmune liver diseases, and multiple sclerosis [25]. Mechanisms that might underlie the immunosuppressive effects include inhibition of Th1- (IFN-γ) and Th17-related (IL-17) cytokines, promotion of Th2-related cytokines (IL-4 and IL-5), release of T_reg_ cell-related cytokines (IL-10 and TGF-β), and the induction of regulatory cells [25], [26]. In particular, the roles of T_reg_ cells have been investigated for their role in host immune regulation of many parasitic infections [4], [8], [9], [27], [28]. In addition, Aranzamendi et al. have reported that *T. spiralis* infection increases the number of T_reg_ cells and that adoptive transfer of CD4^+^ T cells from *T. spiralis*-infected mice suppresses lung inflammation [29]. However, there is little information regarding the direct adoptive transfer of T_reg_ cells isolated from parasite-infected animals and its effects. In the present study, we assessed the functional and molecular characteristics of parasite-induced T_reg_ cells using a mouse model of OVA-alum allergic airway inflammation. Adoptive transfer of Inf(+)Foxp3^+^ cells ameliorated airway inflammation by enhancing T_reg_-cell recruitment around sites of inflammation and thereby inhibiting the Th2 response.

We had three sets of questions regarding parasite-induced T_reg_ cells. First, we asked, do parasite-induced T_reg_ cells have a stronger effect on airway inflammation than natural T_reg_ cells? If so, what are the differences between the two types of T_reg_ cells? To answer these questions, we characterized the T_reg_-cell population (CD4^+^Foxp3^+^ T cells), which included both natural T_reg_ cells (nT_reg_; CD4^+^CD25^+^Foxp3^+^T cells) and inducible T_reg_ cells (iT_reg;_ CD4^+^CD25^-^Foxp3^+^T cells), because *T. spiralis* infection promotes the proliferation and activation of both iT_reg_ and nT_reg_ cells [4]. Several previous studies have shown that both naturally occurring and antigen-driven T_reg_ cells regulate allergen-induced Th2 responses in mice and humans [30]–[32]. In a cockroach allergen-alum model, McGee and Agrawal showed that adoptive transfer of either nT_reg_ cells or iT_reg_ cells reversed airway inflammation and AHR to methacholine; the effect lasted for at least four weeks. In our experiments, although cells from infected and uninfected mice had anti-inflammatory effects, the CD4^+^Foxp3^+^T cells isolated from *T. spiralis*-infected mice [Inf(+)Foxp3^+^] reduced artificially-induced airway inflammation to a greater extent than did CD4^+^Foxp3^+^T cells from uninfected mice [Inf(-)Foxp3^+^] (Fig. 2–5). These results might reflect the activation of CD4^+^Foxp3^+^T cells during *T. spiralis* infection. Inf(+)Foxp3^+^ cells expressed several surface proteins, including CTLA-4 and CD39 (Fig. 8B), which are related to dendritic cell (DC) regulatory functions [10]. CTLA-4 on the surface of T_reg_ cells downregulates or precludes the upregulation of CD80 and CD86, the major co-stimulatory molecules on antigen-presenting cells [10]. Extracellular ATP, an indicator of tissue destruction, exerts inflammatory effects on DCs [10]. As an anti-inflammatory mechanism, catalytic inactivation of extracellular ATP by CD39 on T_reg_ cells might prevent the harmful effects of ATP on DC function [10]. CTLA-4 also mediates T-cell downregulation during chronic filarial infections [9]. In addition, GARP and OX-40, which are expressed on the surface of activated T_reg_ cells and regulate the bioavailability of TGF-β, were highly expressed in Inf(+)Foxp3 cells. GARP potently suppresses the proliferation and differentiation of naive T cells into T effector cells and suppresses IL-2 and IFN-γ production, leading to the differentiation of naive T cells into induced T_reg_ cells [33]. The process is linked to Smad2/3 phosphorylation and is partially suppressed by the inhibition of TGF-β signaling. OX40 (CD137) is also generally expressed on mouse T_reg_ cells. We observed that Inf(+)Foxp3^+^ cells repressed the proliferation of splenocytes, including T cells, to a greater extent than did Inf(-)Foxp3^+^ cells (Fig. 8C). Blocking OX40 on T_reg_ cells with agonist antibodies inhibits the cells' ability to suppress and restores effecter T-cell proliferation [34], [35]. *Capg*, a cancer suppressor gene, is specifically upregulated in T_reg_ cells during chronic helminth infection [36], [37]. We found that *Gzmb* gene expression was upregulated in Inf(+)Foxp3^+^ cells (Fig. 8B). Activated T_reg_ cells also upregulate *Gzmb* expression, and T_reg_ cells kill responder cells via Gzmb-dependent mechanisms [38]. Gzmb-deficient T_reg_ cells have reduced suppressive activity in vitro [10]. Gzmb is released by in vitro-activated T_reg_ cells, and it functionally drives apoptosis in naive B cells [39]. Gzmb is also upregulated in infected animals [37]. We found that OVA-specific serum IgE was reduced in Inf(+)Foxp3^+^ adoptive transfer mice. Thus, in answer to our first questions, these results showed that *T. spiralis*-induced T_reg_ cells are more potent than natural T_reg_ cells.

In our second set of questions, we asked, how do T_reg_ cells regulate airway inflammation? Are they activated in the spleen, at peripherally located lymph nodes, or at inflammatory sites? In this study, although we could not directly evaluate T_reg_ cell population in the lung by FACS analysis because we have technical limitation such for preparation from lung tissue. However, we observed many activated Foxp3-eGFP cells around inflammation sites in the airways, but it was difficult to detect cells in the absence of inflammation (Figs. 5 and 7, S1 and S4 Figs.). We also found that some T_reg_-cell homing receptors, for example, CCR5 and CCR9, were highly expressed in Inf(+)Foxp3^+^ mice, enabling the T_reg_ cells to migrate rapidly to inflammation sites (Fig. 8). Many other homing receptors on T_reg_ cells are involved in the inflammatory recruitment of T_reg_ cells in different immunological settings, including CCR1, CCR2, CCR4, CCR5, CCR8, CCR9, CXCR3, CXCR4, CXCR5, CXCR6, and the P- and E-selectin ligands [40]. T_reg_ cells first migrate from blood to the inflamed allograft, where they are essential for the suppression of inflammation [41]. This process is dependent on the chemokine receptors CCR2, CCR4, and CCR5 and the P- and E-selectin ligands [42]. The absence of CCR5 is associated with impaired recruitment of T_reg_ cells and with decreased IL-10 expression, reflecting the receptor's potent anti-inflammatory activity [42]. Interestingly, levels of CCR9, a gut homing receptor, were higher in Inf(+)Foxp3^+^ cells than in Inf(-)Foxp3^+^ cells. The results suggest that *T. spiralis* stimulates T_reg_-cell recruitment to the intestine during the intestinal phase of infection.

Finally, we asked, is it more effective to transfer T_reg_ cells before or after the induction of inflammation? We addressed this question in two stages. First, we determined the preventive effects of T_reg_-cell transfer [Stage I]. Second, we determined the therapeutic effects of the cells [Stage II]. The results suggest that both methods are effective in this allergic airway inflammation model. Introduction of Inf(+)Foxp3^+^ cells before airway inflammation induction elicited T_reg_-cell recruitment in the spleen and LLN and increased IL-10 production in the LLN (Figs. 3 and 5, S1 Fig.). Introduction of Inf(+)Foxp3^+^ cells during OVA challenge did not elicit T_reg_-cell recruitment in the spleen (Fig. 7). These results indicate that transferred T_reg_ cells can long survive in recipient mice and rapidly migrate to inflammation sites using their activated homing receptors. In this study, more Foxp3 eGFP cells were found in the Inf(+)Foxp3^+^ adoptive transfer mice before allergic inflammation induction (day 0) than in the Inf(+)Foxp3^+^ adoptive transfer mice during OVA challenge (day 17) (Fig. 5 and 7). This phenomenon could not be explained by our present data, the possibility of proliferation of injected the cell in recipient mice will be evaluated in further study.

In conclusion, *T. spiralis* infection promotes the proliferation and functional activation of T_reg_ cells, which migrate to inflammation sites and suppress the immune response more effectively than non-parasite-induced T_reg_ cells. The adoptive transfer of Inf(+)Foxp3^+^ cells is an effective method for the prevention of allergic airway inflammation in mice and is a promising approach for the treatment of allergic airway diseases.

## Supporting Information

S1 Fig **Analysis of** ***Foxp3*** **gene expression and CTLA4^+^Foxp3^+^ cells in the lung of the CD4^+^Foxp3^+^T cell adoptive transferred mice before asthma induction.** (Stage I). Total RNA was isolated from the lung tissue of each mouse, and cDNA was synthesized according to the manufacturer's protocol. The gene expression levels of Foxp3 in the lungs of each group were analyzed using real-time PCR. The GAPDH gene was used as a control. Data are representative of three independent experiments (A). Paraffin sections of lungs from all of mice (6 mice/group) receiving CD4^+^Foxp3^+^T cells were immunofluorescently stained for CTLA-4, and nuclei (DAPI) representative pictures (merge of CTLA-4, GFP, and DAPI filed screens per high power filed) are shown (white bar = 100 µm). These figures were used for the analysis of population of CTLA4^+^Foxp3^+^ cells in lung tissue by Image J program, we calculated the number of the CTLA4^+^Foxp3^+^ cells per total 10000 DAPI^+^ cells (B) (The result was shown at Fig. 5D in main text). OVA-; PBS treated mice, OVA+; allergic airway inflammation-induced mice, IV(inf)+(-); CD4^+^Foxp3^+^T cell of normal mice adoptive transferred mice, IV(inf)+(+); CD4^+^Foxp3^+^T cell of *T. spiralis-infected* mice adoptive transferred mice, a; *p<0.05, **p<0.01, *n* = 6 mice/group, 3 independent experiments].
(PPTX)

S2 Fig **Amelioration of airway inflammation with CD4^+^Foxp3^+^T cell adoptive transfer during asthma induction (Stage II).** The histological appearance of lungs after challenge with OVA and cell transfer (bar = 50 µm). The thin sections of lung were stained with hematoxylin-eosin (H&E) and PAS stains (A). Relative quantification of *eotaxin* gene expression in lung after the induction of airway inflammation. Total RNA was extracted from lung tissue and cDNA was synthesized. The gene expression levels of *eotaxin* in the lungs of each group were analyzed using real-time PCR. The GAPDH gene was used as a control. (B). The number of inflammatory cells in the BALF samples was counted after Diff-Quik staining (C). [OVA-; PBS treated mice, OVA+; allergic airway inflammation-induced mice, IV(inf)+(+); CD4^+^Foxp3^+^T cell of *T. spiralis-infected* mice adoptive transferred mice, IV(inf)+(-); CD4^+^Foxp3^+^T cell of normal mice adoptive transferred mice, *p<0.05, **p<0.01, *n* = 6 mice/group, 3 independent experiments].
(PPTX)

S3 Fig **Concentration of cytokines in BALF and OVA specific cytokines of lymphocytes isolated from lung draining lymph node.** IFN-γ and IL-10 concentrations were measured in the BALF samples (A). The lymphocytes were activated by OVA. The wells were incubated with 1 µg/mL of OVA for 16 h at 4°C, and then the lymphocytes isolated from lung draining lymph node were added and incubated for three days. After activation, the concentrations of cytokines in the supernatant were measured using ELISA kits, in accordance with the manufacturer's instructions (B). [OVA-; PBS treated mice, OVA+; allergic airway inflammation-induced mice, IV(inf)+(+); CD4^+^Foxp3^+^T cell of *T. spiralis-infected* mice adoptive transferred mice, IV(inf)+(-); CD4^+^Foxp3^+^T cell of normal mice adoptive transferred mice, *p<0.05, **p<0.01, *n* = 6 mice/group, 3 independent experiments].
(PPTX)

S4 Fig **Analysis of CTLA4^+^Foxp3^+^ cells in the lung of the CD4^+^Foxp3^+^T cell adoptive transferred mice before asthma induction.** (Stage II). Paraffin sections of lungs from all of mice (6 mice/group) receiving CD4^+^Foxp3^+^T cells were immunofluorescently stained for CTLA-4, and nuclei (DAPI) representative pictures (merge of CTLA-4, GFP, and DAPI filed screens per high power filed) are shown (white bar = 100 µm). These figures were used for the analysis of population of CTLA4^+^Foxp3^+^ cells in lung tissue by Image J program, we calculated the number of the CTLA4^+^Foxp3^+^ cells per total 1×10^5^ DAPI positive cells. (The result was shown at Fig. 7C in main text). OVA-; PBS treated mice, OVA+; allergic airway inflammation-induced mice, IV(inf)+(-); CD4^+^Foxp3^+^T cell of normal mice adoptive transferred mice, IV(inf)+(+); CD4^+^Foxp3^+^T cell of *T. spiralis-infected* mice adoptive transferred mice, a; *p<0.05, **p<0.01, *n* = 6 mice/group, 3 independent experiments].
(PPTX)

S1 Table **Primers used for real-time PCR.**
(DOCX)
