# Supplementary material for: Parasitic Nematode-Induced CD4+Foxp3+T Cells Can Ameliorate Allergic Airway Inflammation
Source: PLoS Negl Trop Dis. 2014 Dec 18;8(12):e3410. doi: 10.1371/journal.pntd.0003410 (PMC4270642; doi:10.1371/journal.pntd.0003410)
Supplement: S1 Fig — Analysis of Foxp3 gene expression and CTLA4+Foxp3+ cells in the lung of the CD4+Foxp3+T cell adoptive transferred mice before asthma induction. (Stage I). Total RNA was isolated from the lung tissue of each mouse, and cDNA was synthesized according to the manufacturer's protocol. The gene expression levels of Foxp3 in the lungs of each group were analyzed using real-time PCR. The GAPDH gene was used as a control. Data are representative of three independent experiments (A). Paraffin sections of lungs from all of mice (6 mice/group) receiving CD4+Foxp3+T cells were immunofluorescently stained for CTLA-4, and nuclei (DAPI) representative pictures (merge of CTLA-4, GFP, and DAPI filed screens per high power filed) are shown (white bar = 100 µm). These figures were used for the analysis of population of CTLA4+Foxp3+ cells in lung tissue by Image J program, we calculated the number of the CTLA4+Foxp3+ cells per total 10000 DAPI+ cells (B) (The result was shown at Fig. 5D in main text). OVA-; PBS treated mice, OVA+; allergic airway inflammation-induced mice, IV(inf)+(-); CD4+Foxp3+T cell of normal mice adoptive transferred mice, IV(inf)+(+); CD4+Foxp3+T cell of T. spiralis-infected mice adoptive transferred mice, a; *p<0.05, **p<0.01, n = 6 mice/group, 3 independent experiments]. (PPTX) [file pntd.0003410.s001.pptx]

## Slide 1
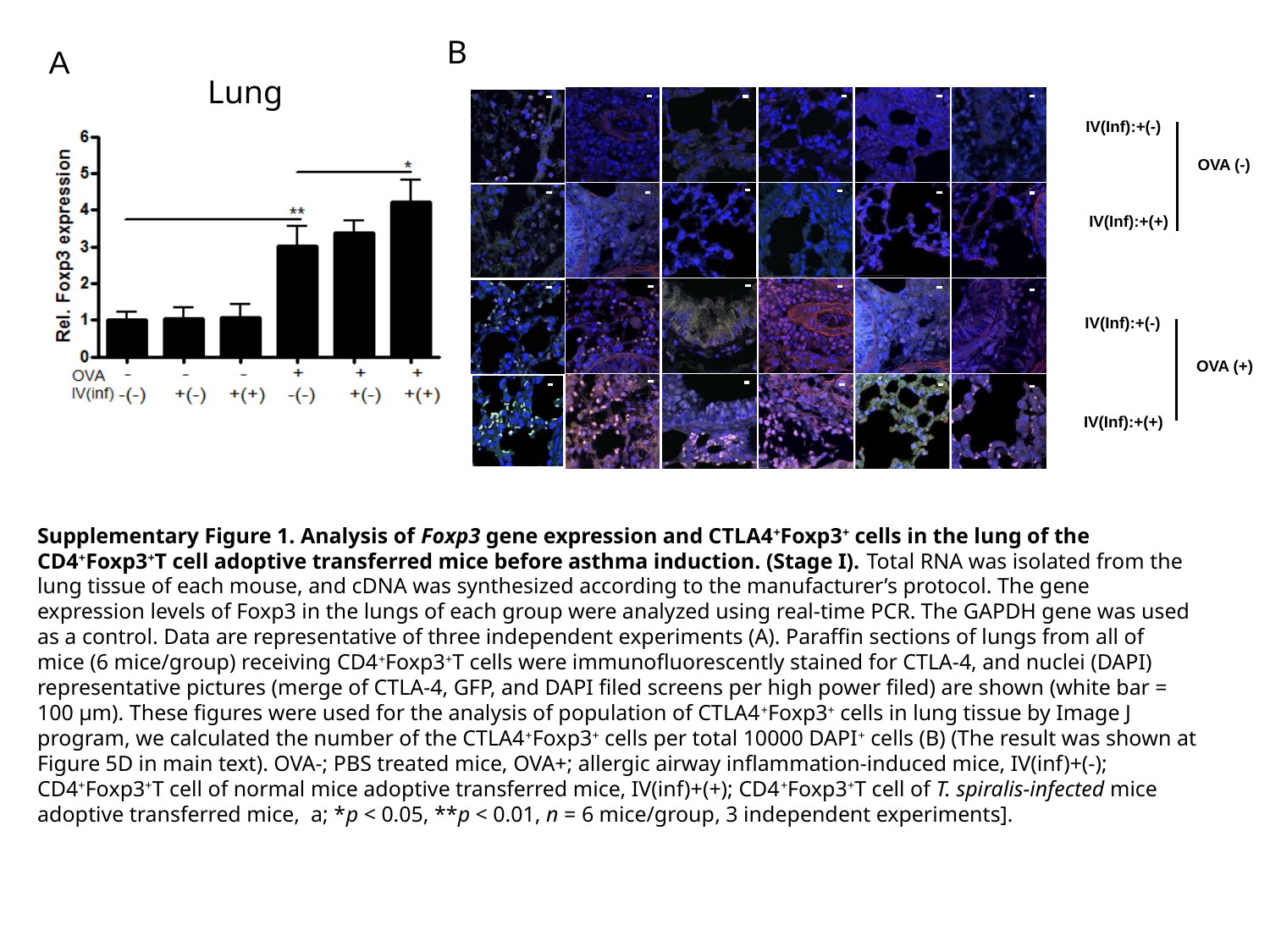

B
IV(Inf):+(-)
OVA (-)
IV(Inf):+(+)
IV(Inf):+(-)
OVA (+)
IV(Inf):+(+)
A
Lung
Supplementary Figure 1. Analysis of Foxp3 gene expression and CTLA4+Foxp3+ cells in the lung of the CD4+Foxp3+T cell adoptive transferred mice before asthma induction. (Stage I). Total RNA was isolated from the lung tissue of each mouse, and cDNA was synthesized according to the manufacturer’s protocol. The gene expression levels of Foxp3 in the lungs of each group were analyzed using real-time PCR. The GAPDH gene was used as a control. Data are representative of three independent experiments (A). Paraffin sections of lungs from all of mice (6 mice/group) receiving CD4+Foxp3+T cells were immunofluorescently stained for CTLA-4, and nuclei (DAPI) representative pictures (merge of CTLA-4, GFP, and DAPI filed screens per high power filed) are shown (white bar = 100 µm). These figures were used for the analysis of population of CTLA4+Foxp3+ cells in lung tissue by Image J program, we calculated the number of the CTLA4+Foxp3+ cells per total 10000 DAPI+ cells (B) (The result was shown at Figure 5D in main text). OVA-; PBS treated mice, OVA+; allergic airway inflammation-induced mice, IV(inf)+(-); CD4+Foxp3+T cell of normal mice adoptive transferred mice, IV(inf)+(+); CD4+Foxp3+T cell of T. spiralis-infected mice adoptive transferred mice, a; *p < 0.05, **p < 0.01, n = 6 mice/group, 3 independent experiments].
