# Supplementary material for: Parasitic Nematode-Induced CD4+Foxp3+T Cells Can Ameliorate Allergic Airway Inflammation
Source: PLoS Negl Trop Dis. 2014 Dec 18;8(12):e3410. doi: 10.1371/journal.pntd.0003410 (PMC4270642; doi:10.1371/journal.pntd.0003410)
Supplement: S2 Fig — Amelioration of airway inflammation with CD4+Foxp3+T cell adoptive transfer during asthma induction (Stage II). The histological appearance of lungs after challenge with OVA and cell transfer (bar = 50 µm). The thin sections of lung were stained with hematoxylin-eosin (H&E) and PAS stains (A). Relative quantification of eotaxin gene expression in lung after the induction of airway inflammation. Total RNA was extracted from lung tissue and cDNA was synthesized. The gene expression levels of eotaxin in the lungs of each group were analyzed using real-time PCR. The GAPDH gene was used as a control. (B). The number of inflammatory cells in the BALF samples was counted after Diff-Quik staining (C). [OVA-; PBS treated mice, OVA+; allergic airway inflammation-induced mice, IV(inf)+(+); CD4+Foxp3+T cell of T. spiralis-infected mice adoptive transferred mice, IV(inf)+(-); CD4+Foxp3+T cell of normal mice adoptive transferred mice, *p<0.05, **p<0.01, n = 6 mice/group, 3 independent experiments]. (PPTX) [file pntd.0003410.s002.pptx]

## Slide 1
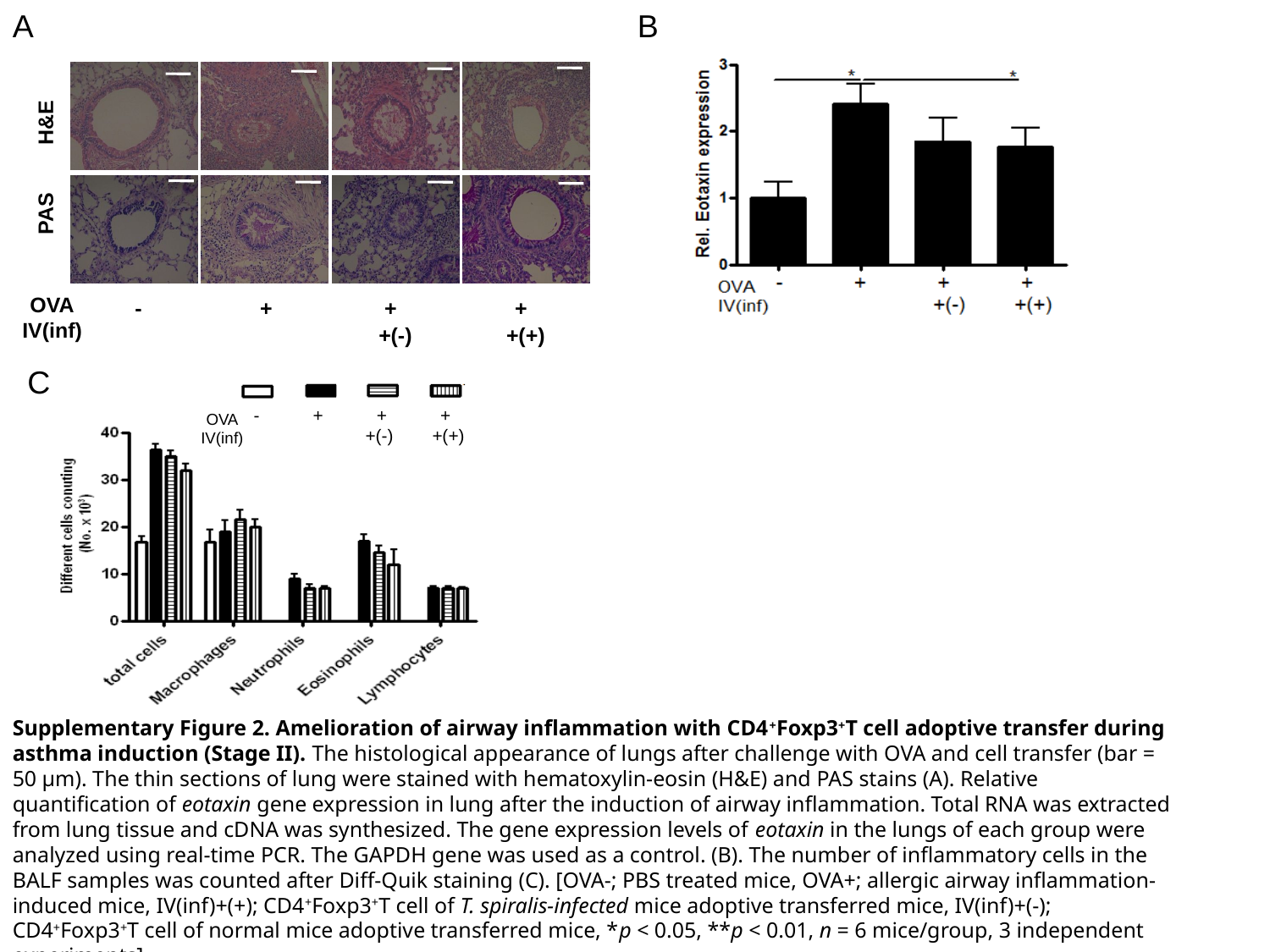

A
 PAS H&E
OVA
IV(inf)
 - + + +
 +(-) +(+)
B
C
 - + + +
OVA
IV(inf)
 +(-) +(+)
Supplementary Figure 2. Amelioration of airway inflammation with CD4+Foxp3+T cell adoptive transfer during asthma induction (Stage II). The histological appearance of lungs after challenge with OVA and cell transfer (bar = 50 µm). The thin sections of lung were stained with hematoxylin-eosin (H&E) and PAS stains (A). Relative quantification of eotaxin gene expression in lung after the induction of airway inflammation. Total RNA was extracted from lung tissue and cDNA was synthesized. The gene expression levels of eotaxin in the lungs of each group were analyzed using real-time PCR. The GAPDH gene was used as a control. (B). The number of inflammatory cells in the BALF samples was counted after Diff-Quik staining (C). [OVA-; PBS treated mice, OVA+; allergic airway inflammation-induced mice, IV(inf)+(+); CD4+Foxp3+T cell of T. spiralis-infected mice adoptive transferred mice, IV(inf)+(-); CD4+Foxp3+T cell of normal mice adoptive transferred mice, *p < 0.05, **p < 0.01, n = 6 mice/group, 3 independent experiments].
