# Supplementary material for: Parasitic Nematode-Induced CD4+Foxp3+T Cells Can Ameliorate Allergic Airway Inflammation
Source: PLoS Negl Trop Dis. 2014 Dec 18;8(12):e3410. doi: 10.1371/journal.pntd.0003410 (PMC4270642; doi:10.1371/journal.pntd.0003410)
Supplement: S3 Fig — Concentration of cytokines in BALF and OVA specific cytokines of lymphocytes isolated from lung draining lymph node. IFN-γ and IL-10 concentrations were measured in the BALF samples (A). The lymphocytes were activated by OVA. The wells were incubated with 1 µg/mL of OVA for 16 h at 4°C, and then the lymphocytes isolated from lung draining lymph node were added and incubated for three days. After activation, the concentrations of cytokines in the supernatant were measured using ELISA kits, in accordance with the manufacturer's instructions (B). [OVA-; PBS treated mice, OVA+; allergic airway inflammation-induced mice, IV(inf)+(+); CD4+Foxp3+T cell of T. spiralis-infected mice adoptive transferred mice, IV(inf)+(-); CD4+Foxp3+T cell of normal mice adoptive transferred mice, *p<0.05, **p<0.01, n = 6 mice/group, 3 independent experiments]. (PPTX) [file pntd.0003410.s003.pptx]

## Slide 1
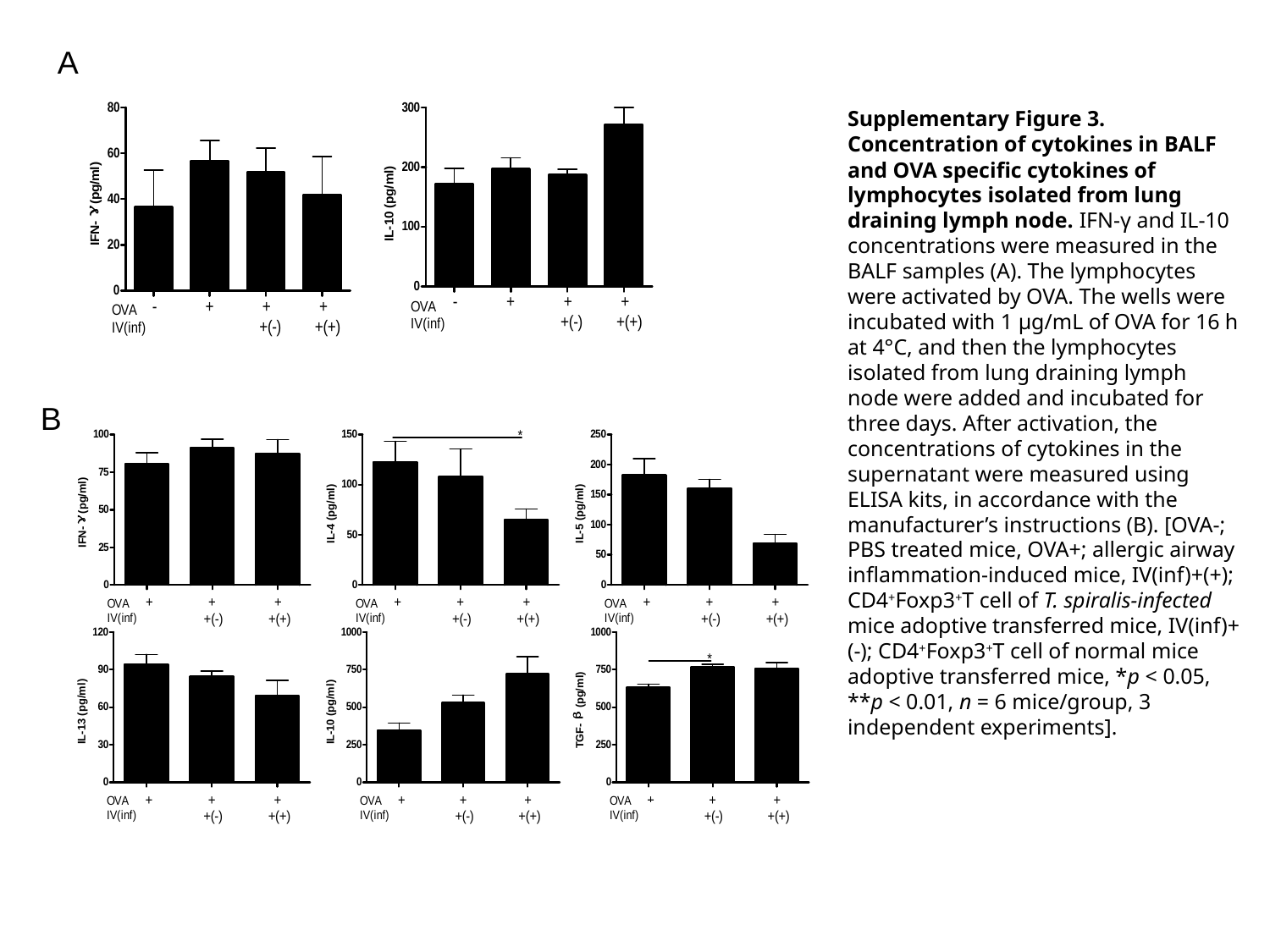

A
Supplementary Figure 3. Concentration of cytokines in BALF and OVA specific cytokines of lymphocytes isolated from lung draining lymph node. IFN-γ and IL-10 concentrations were measured in the BALF samples (A). The lymphocytes were activated by OVA. The wells were incubated with 1 µg/mL of OVA for 16 h at 4°C, and then the lymphocytes isolated from lung draining lymph node were added and incubated for three days. After activation, the concentrations of cytokines in the supernatant were measured using ELISA kits, in accordance with the manufacturer’s instructions (B). [OVA-; PBS treated mice, OVA+; allergic airway inflammation-induced mice, IV(inf)+(+); CD4+Foxp3+T cell of T. spiralis-infected mice adoptive transferred mice, IV(inf)+(-); CD4+Foxp3+T cell of normal mice adoptive transferred mice, *p < 0.05, **p < 0.01, n = 6 mice/group, 3 independent experiments].
B
