# Supplementary material for: Parasitic Nematode-Induced CD4+Foxp3+T Cells Can Ameliorate Allergic Airway Inflammation
Source: PLoS Negl Trop Dis. 2014 Dec 18;8(12):e3410. doi: 10.1371/journal.pntd.0003410 (PMC4270642; doi:10.1371/journal.pntd.0003410)
Supplement: S4 Fig — Analysis of CTLA4+Foxp3+ cells in the lung of the CD4+Foxp3+T cell adoptive transferred mice before asthma induction. (Stage II). Paraffin sections of lungs from all of mice (6 mice/group) receiving CD4+Foxp3+T cells were immunofluorescently stained for CTLA-4, and nuclei (DAPI) representative pictures (merge of CTLA-4, GFP, and DAPI filed screens per high power filed) are shown (white bar = 100 µm). These figures were used for the analysis of population of CTLA4+Foxp3+ cells in lung tissue by Image J program, we calculated the number of the CTLA4+Foxp3+ cells per total 1×10^5^ DAPI positive cells. (The result was shown at Fig. 7C in main text). OVA-; PBS treated mice, OVA+; allergic airway inflammation-induced mice, IV(inf)+(-); CD4+Foxp3+T cell of normal mice adoptive transferred mice, IV(inf)+(+); CD4+Foxp3+T cell of T. spiralis-infected mice adoptive transferred mice, a; *p<0.05, **p<0.01, n = 6 mice/group, 3 independent experiments]. (PPTX) [file pntd.0003410.s004.pptx]

## Slide 1
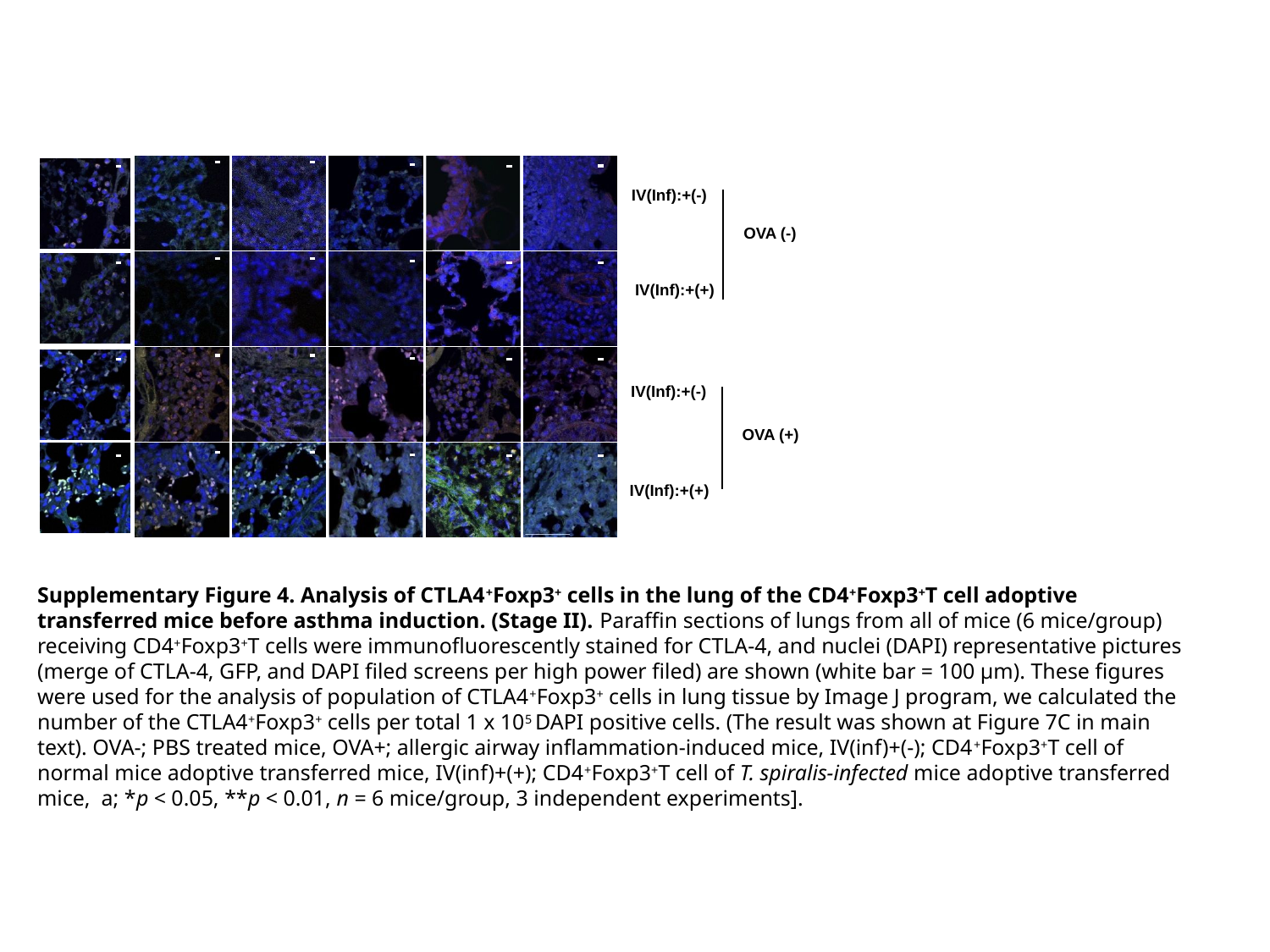

IV(Inf):+(-)
OVA (-)
IV(Inf):+(+)
IV(Inf):+(-)
OVA (+)
IV(Inf):+(+)
Supplementary Figure 4. Analysis of CTLA4+Foxp3+ cells in the lung of the CD4+Foxp3+T cell adoptive transferred mice before asthma induction. (Stage II). Paraffin sections of lungs from all of mice (6 mice/group) receiving CD4+Foxp3+T cells were immunofluorescently stained for CTLA-4, and nuclei (DAPI) representative pictures (merge of CTLA-4, GFP, and DAPI filed screens per high power filed) are shown (white bar = 100 µm). These figures were used for the analysis of population of CTLA4+Foxp3+ cells in lung tissue by Image J program, we calculated the number of the CTLA4+Foxp3+ cells per total 1 x 10^5^ DAPI positive cells. (The result was shown at Figure 7C in main text). OVA-; PBS treated mice, OVA+; allergic airway inflammation-induced mice, IV(inf)+(-); CD4+Foxp3+T cell of normal mice adoptive transferred mice, IV(inf)+(+); CD4+Foxp3+T cell of T. spiralis-infected mice adoptive transferred mice, a; *p < 0.05, **p < 0.01, n = 6 mice/group, 3 independent experiments].
