# Supplementary material for: Parasitic Nematode-Induced CD4+Foxp3+T Cells Can Ameliorate Allergic Airway Inflammation
Source: PLoS Negl Trop Dis. 2014 Dec 18;8(12):e3410. doi: 10.1371/journal.pntd.0003410 (PMC4270642; doi:10.1371/journal.pntd.0003410)
Supplement: S1 Table — Primers used for real-time PCR. (DOCX) [file pntd.0003410.s005.docx]

Table 1. Primers used for real-time PCR

| Primer | Sequence |
| --- | --- |
| GAPDH-for^*^ | 5′ - TACCCCCAATGTGTCCGTC - 3′ |
| GAPDH-rev^†^ | 5′ - AAGAGTGGGAGTTGCTGTTGAAG - 3′ |
| MUC2-for | 5′ –TGT GGC CTG TGT GGG AAC TTT- 3′ |
| MUC2-rev | 5′ –CAT AGA GGG CCT GTC CTC AGG- 3′ |
| MUC5-for | 5′ -TCCGGCTCATCTTCTTCC- 3′ |
| MUC5-rev | 5′ –ACTTGGGCACTGGTGCTG- 3′ |
| Eotaxin-for | 5′- GCGCTTCTATTCCTGCTGCTCACGG- 3′- |
| Eotaxin-ref | 5′- GTGGCATCCTGGACCCACTTCTTC- 3′- |
| CXCR3-for | 5′ –AGA ATC ATC CTG GTC TGA GAC AA- 3′ |
| CXCR3-rev | 5′ –AAG ATA GGG CAT GGC AGC TA- 3′ |
| CTLA-4-for | 5′ –GGA CGC AGA TTT ATG TCA TTG ATC- 3′ |
| CTLA-4-rev | 5′ –CCA AGC TAA CTG CGA CAA GGA- 3′ |
| CCR4-for | 5′ –ATC GTG CAC GCG GTA TTC TCC- 3′ |
| CCR4-rev | 5′ –GAC GGG GTT AAG GCA GCA GTG A- 3′ |
| CCR5- for | 5′ –GGA TTT TCA AGG GTC AGT TC- 3′ |
| CCR5-rev | 5′ –AAC CTT CTT TCT GAG ATC TGG- 3′ |
| CCR9- for | 5′ –ATT GCA CAA GAG TGA AGA CC- 3′ |
| CCR9-rev | 5′ –GTC AAC AGC CTG CAC TAC AA- 3’ |
| CCR10- for | 5′ –CGG AGA AAC CCT TGT AGC CAG- 3′ |
| CCR10-rev | 5′ –GGC CAA GAC TAG GCC ATT GCC- 3′ |
| Tbet- for | 5′ –CAA CAA CCC CTT TGC CAA AG- 3′ |
| Tbet-rev | 5′ –TCC CCC AAG CAG TTG ACA GT- 3′ |
| GATA3- for | 5′ –CAT TAC CAC CTA TCC GCC CTA TG- 3′ |
| GATA3-rev | 5′ –CAC ACA CTC CCT GCC TTC TGT- 3′ |
| RORλ- for | 5′ –CAC GGC CCT GGT TCT CAT- 3′ |
| RORλ-rev | 5′ –CAG ATG TTC CAC TCT CCT CTT CTC T- 3′ |
| Foxp3-for | 5′ –GGC CCT TCT CCA GGA CAG A- 3′ |
| Foxp3-rev | 5′ –GCT GAT CAT GGC TGG GTT GT- 3′ |
| CD62L-for | 5′ –CAT TCC TGT AGC CGT CAT GG- 3′ |
| CD62L-rev | 5′ –AGG AGG AGC TGT TGG TCA TG- 3′ |
| OX40-for | 5′ –TAT GGT GAG CCG CTG TGA TC- 3′ |
| OX40-rev | 5′ –ACA GTC AAG GGA GCC AGC AG- 3′ |
| Klrg1- for | 5′ –GGC TTG AGG AAC ATT GAT GG- 3′ |
| Klrg1-rev | 5′ –TCA AGC TGT TGG TAA GAA TCC TC- 3′ |
| Capg- for | 5′ –GCT GTG TGG CAA AAT CTA CAT C- 3′ |
| Capg- rev | 5′ –GAT GAA GCC ATC AGC CAC TT- 3′ |
| Gzmb- for | 5′ –GCT GCT CAC TGT GAA GGA AGT- 3′ |
| Gzmb- rev | 5′ –TGG GGA ATG CAT TTT ACC AT- 3′ |

^*^for; forward

^†^rev; reverse
